# Rat Bone Mesenchymal Stem Cell-Derived Exosomes Loaded with miR-494 Promoting Neurofilament Regeneration and Behavioral Function Recovery after Spinal Cord Injury

**DOI:** 10.1155/2021/1634917

**Published:** 2021-10-01

**Authors:** Wei Huang, Miaoman Lin, Cunheng Yang, Fumin Wang, Meng Zhang, Junxiao Gao, Xiaobing Yu

**Affiliations:** ^1^Department of Orthopaedics, Affiliated Zhongshan Hospital of Dalian University, Dalian 116001, China; ^2^Department of Orthopaedics, Dongguan Tungwah Hospital, Dongguan 523000, China

## Abstract

Exosomes (Exo) exhibit numerous advantages (e.g., good encapsulation, high targeting efficiency, and easy to penetrate the blood-brain barrier to the central nervous system). Exosomes are recognized as prominent carriers of mRNAs, siRNAs, miRNAs, proteins, and other bioactive molecules. As confirmed by existing studies, miR-494 is important to regulate the occurrence, progression, and repair of spinal cord injury (SCI). We constructed miR-494-modified exosomes (Exo-miR-494). As indicated from related research in vitro and vivo, Exo-miR-494 is capable of effectively inhibiting the inflammatory response and neuronal apoptosis in the injured area, as well as upregulating various anti-inflammatory factors and miR-494 to protect neurons. Moreover, it can promote the regeneration of the neurofilament and improve the recovery of behavioral function of SCI rats.

## 1. Introduction

Spinal cord injury (SCI) refers to a catastrophic event in spinal surgery, commonly leading to severe neurological and sensory dysfunction [1]. It still lacks effective treatment measures in clinical practice, imposing huge psychological and economic burden to patients and the country's medical services. Over the past few years, the powerful proliferation and differentiation potential of mesenchymal stem cells (MSCs) has achieved new progress for treating SCI [2]. However, the transplanted MSCs have numerous defects (e.g., low survival rate, carcinogenicity, and poor targeting) [3]. As proven by numerous studies, the therapeutic effect of MSCs may be primarily attributed to the secreted exosomes (Exo) [4].

Exos are small membrane vesicles originating from intracellular corpuscles, exhibiting a diameter of about 30–200 nm, which are packed with various proteins, lipids, and RNA. These vesicles can be released into extracellular fluid, blood, and cerebrospinal fluid by cells of all living systems [5, 6]. Under physiological and pathophysiological conditions, they are capable of transferring their cargo between source cells and target cells via extracellular fluid, blood, and cerebrospinal fluid, and they critically impact intercellular communication [7]. Compared with the transplanted MSCs, MSCs-Exo exhibit the advantages of long stay time in the body, low carcinogenicity, and high targeting efficiency, as well as low immune rejection and easy of passing through the blood-brain barrier. Thus, it acts as an excellent carrier of foreign small molecule drugs.

Exosomes are closely related to the occurrence, progress, and recovery of central nervous system injury [8–10]. After spinal cord injury, exosomes can regulate the autonomic regeneration function of neurons and, also, affect the remyelination of the spinal cord, the metabolic activity, and inflammatory environment around the lesions. Exosomes can also actively mobilize the internal regeneration ability of neurons and reduce the adverse factors of the external surrounding microenvironment [11]. Studies have shown that bone marrow mesenchymal stem cell-exosomes can protect the injured spinal cord by inhibiting pericyte pyroptosis [12]. Furthermore, nanoscale exosomes secreted by stem cells have great potential in reducing apoptosis and inflammation, enhancing angiogenesis, and improving functional and behavioral recovery after spinal cord injury [13].

MicroRNAs (miRNAs) are small noncoding RNA molecules, capable of regulating various biological activities and profoundly impacting cell proliferation, differentiation, aging, death, neural activity, etc. miRNAs show a close relationship to the pathological processes of spinal cord injury (e.g., inflammatory reaction, demyelination, oxidative stress, and neuronal apoptosis) [14].

A large number of miRNAs have been found in the central nervous system of mammals, which play an important role in neurogenesis, development, and regeneration [15]. As reported by Xu et al., in the treatment of spinal cord injury, the overexpressions of miR-124 and miR-133b promote the differentiation of neural stem cells [16, 17]. It has been reported that miR-494 can reduce autophagy and apoptosis of neurons after SCI by targeting IL-13 [18]. In addition, Wang et al. suggested that miR-494 is capable of regulating the effects of the Nogo/NgR signaling pathway on astrocyte proliferation and synaptic remodeling after SCI [19].

Though miR-494 exhibits a strong repair ability for SCI, the amount of secretion by living cells is low, and the efficiency of in vivo transportation is limited, thereby adversely affecting its therapeutic effect. This study planned to load exogenous miR-494 into exosomes through reagent transfection to determine whether exosomes can effectively deliver miR-494 to the spinal cord lesion, as well as verify the repair effect of Exo^miR-494^ on SCI rats.

## 2. Methods and Materials

### 2.1. Experimental Design

**Figure 1:**
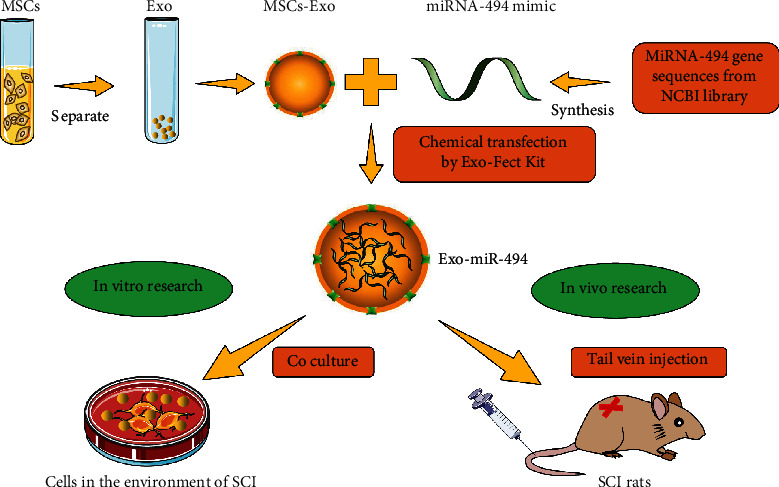
The flow chart of the whole experiment.

The whole experiment is divided into three parts, “construction of Exo^miR-494^/Exo-miR-494,” “in vitro research,” and “in vivo research.” As shown in Figure 1, firstly, we used the kit to extract the exosomes from rat MSCs and then designed and synthesized the miR-494 mimics according to the gene sequence of miR-494. Next, we used the Exo-Fect™ transfection kit to transfect miR-494 into the MSCs-Exo and got the Exo^miR-494^. In the second part, we cocultured the constructed Exo-miR-494 with dorsal root ganglions (DRGs) and rat NR8383 macrophages in the spinal cord injury environment to study whether it has antiapoptotic and anti-inflammatory effects. Finally, we conducted animal experiments. We injected Exo^miR-494^ into SCI rats by tail vein injection to further study its effects on motor and behavioral functions of SCI rats.

### 2.2. Animals

The experimental procedures were approved by the Affiliated Zhongshan Hospital of Dalian University Science Review Committee and Animal Research Ethics Review Committee and conducted in line with the National Institutes of Health Guide for the Care and Use of Laboratory Animals.

First, we will use 2-week-old SD rats to extract bone MSCs. Then, in order to ensure that the grouping is statistically significant, we also need 6 SCI rats for the in vitro organ imaging experiment. Next, we need 6 normal healthy rats and 6 SCI rats for the drug toxicity test (the same animal can take the lung and liver at the same time, as well as the determination of lactate dehydrogenase, superoxide dismutase, and alanine aminotransferase in the liver).

Next, we carried out the animal experiment to verify the therapeutic effect of Exo^miR-494^ on spinal cord injury. On the whole, grouping of animal experiments, i.e., the Control group (rats were treated with PBS after SCI), Sham group (rats were treated with PBS after sham operation), Exo-miR-con group (rats were treated with pure exosome after SCI), and Exo-miR-494 group (rats were treated with Exo^miR-494^ after SCI). In order to ensure the statistical significance of each experiment and ensure that the number of rats in each group is 6, we divided the animals into the Control group (*n* = 6), Sham group (*n* = 6), Exo-miR-con group (*n* = 6), and Exo-miR-494 group (*n* = 6) to form a large group. In this way, we need a large group of animals to determine the relative expression of miR-494 and growth-associated protein-43(GAP-43) in vivo. Similarly, we need a large group of animals for the hematoxylin-eosin (HE) experiment, immunofluorescence analysis experiment, locomotor capacity assessment experiment, Paw Withdraw Thermal Latency (PWTL) experiment, and hind leg neurophysiological experiment. Statistically, we need 2 2-week-old SD rats, 6 normal healthy rats, 42 sham rats, and 138 SCI rats (excluding the rats with SCI model failure or death, continue to supplement the above conditions, and all animal numbers are confirmed by the Animal Research Ethics Review Committee).

### 2.3. Extraction and Identification of Bone Marrow MSCs

Firstly, male SD rats (80-100 g) of 2 weeks old were prepared, the whole bone marrow culture method was used to extract MSCs, and then, MSCs were cultured in primary culture until the cell density reached 90%. Subsequently, the serum-free FBS (Gibco™, USA) was used for 48 h expansion culture in the complete medium to obtain about 300 mL of stem cell culture supernatant for subsequent experiments.

Next, we used flow cytometry to identify the expression of the bone marrow MSC marker protein. In short, firstly, MSCs were digested with trypsin and mixed with 100 *μ*L PBS, and then, 10 *μ*L of CD29, CD45, and CD90 antibodies (eBioscience, USA) was added. The mixture was wrapped with tin foil and incubated at 4°C for 30 min. After incubation, centrifuge for 5 min, clean 3 times, and resuspend MSCs with 500 *μ*L PBS. Finally, the marker protein was detected by flow cytometry (Cytek, USA), and the data were processed by FlowJo 8.7 software.

### 2.4. Preparation and Identification of Exosomes Derived from Bone Marrow MSCs

With the kit method, the exosomes were separated from the collected cell supernatant. In brief, the cell supernatant was placed at 4°C and then centrifuged at 3000 g for 10 min to remove excess cell debris. Subsequently, it was centrifuged to remove impurities. GS™ Exosome Isolation Reagent (for serum or plasma, Geneseed Biotech Co., China) was added to the supernatant, and the instructions (Supplement 1) were complied with to extract exosomes from the extracellular supernatant. In summary, 500 *μ*L of the rat supernatant was extracted, and 1/3 times the volume of the supernatant was added to the solution A. And the mixed solution was left to stand at 4°C for 15 min. At ambient temperature, centrifugation was conducted again at 13,000 g for 10 min, and the supernatant was aspirated; the solution was centrifuged at 13,000 g for 30 s, and the residual supernatant was aspirated. Lastly, the identical volume of B solution was added as Reagent A, and the solution was mixed by pipetting and centrifuged at 13,000 g. Finally, the supernatant was aspirated and removed; afterwards, we used bicinchoninic acid (BCA) to detect the protein concentration in the exosome and then resuspended it in PBS with a total protein concentration of 10 *μ*g/*μ*L [20]. The resulting precipitate was an exosome, which was stored at -80°C. The mentioned method was repeated to extract all the exosomes in the supernatant of 500 mL for subsequent cell and animal experiments.

Next, we used the following methods to identify exosomes.

#### 2.4.1. Transmission Electron Microscopy (TEM)

Take out 5 *μ*L exosomes, add 5 *μ*L PBS, then add the mixture to the copper wire, and stand for 1 min; next, 10 *μ*L phosphotungstic acid was added to the copper mesh and stood for 1 min to remove the liquid suspended on the surface (refer to Supplement 2 for specific methods). Finally, the imaging was detected by 80 kV transmission electron microscopy (Hitachi, JAPAN).

#### 2.4.2. Nanoparticle Tracking Analysis (NTA)

Take 10 *μ*L exosomes and add 990 *μ*L PBS which was mixed evenly, and nanoparticle tracking analysis instrument (Particle Metrix, Germany) was used to detect the sample. Besides, we detected the marker proteins CD9 and CD63 of exosomes by western blot (WB) analysis (see “WB Analysis” for specific methods).

### 2.5. Preparation of miR-494 Mimic and Negative Controls (NC)

By complying with the instructions, miR-494 mimics and miR-494 NC were synthesized by Invitrogen (GenePharma, Shanghai, China), treated with diethyl pyrocarbonate (DEPC), and then placed and stored at a low temperature and in a dark place. The oligonucleotides used in this sequence are shown in Table 1.

### 2.6. MiR-494 Was Loaded into Exosomes by Chemical Transfection

As mentioned above [21], we used Exo-Fect™ (System Biosciences, Palo Alto, USA) to load miR-494. First, miR-494 (100-300 pmol), exosomes (200 *μ*g in 50 *μ*L), and Exo-Fect were mixed and incubated at 37°C for 20 min (for the Control group, only miR-494 and exosomes were mixed without adding Exo-Fect), then ExoQuick-TC reagent (System Biosciences, Palo Alto, USA) was used to culture the mixture on ice for 20 min. Finally, exosomes/Exo^miR-494^ were centrifuged at 13,000 × g for 5 min to collect and resuspend in phosphate buffer saline (PBS, Gibco™, USA) for reverse transcription (qPCR).

### 2.7. Quantitative Determination and Antidegradation Test of Exo^miR-494^

#### 2.7.1. Exo^miR-494^ Loading Capacity

5′-32P were used to label miR-494. The loading capacity of exosomes was determined by the radioactivity in the supernatant and then quantified with a Packard instant imager. The loading rate was calculated using the following equation: the miR − 494 loading rate = (CPM in pellet/CPM in supernatant) × 100. Moreover, the loading capacity of Exo^miR-494^ was calculated by the aforesaid formula.

#### 2.7.2. Exo^miR-494^ Antidegradation Test

Exo^miR-494^ were incubated with 500 *μ*L RNase A with various concentrations (0.01 *μ*g/mL, Solarbio, USA). After being incubated at 37°C for 0, 30, 60, and 90 min, naked miR-494 mimic was classified as the control. Moreover, the undegraded miR-494 was quantitatively analyzed by RT-PCR.

### 2.8. Apoptosis Test

Rat dorsal root ganglion cells (DRGs) were obtained from the cell bank center of the Shanghai Academy of Biological Sciences (SIBS) and were seeded in a 6-well plate with 1 × 10^6^ cells/well. When DRGs fused more than 90%, the SCI environment was simulated with 10 mg lipopolysaccharide (LPS, YT1319, YITA, China) and hypoxia (2% O_2_) for 24 h. Referring to previous studies [20], next, the experimental groups were as follows (*n* = 3): Control group (0.5 mL PBS), Exo-miR-Con group (100 *μ*g pure exosomes in 0.5 mL PBS), and Exo-miR-494 group (100 *μ*g Exo^miR-494^ in 0.5 mL PBS). After 24 hours of coculture, DRGs were washed twice with PBS, and the survival of the cells was evaluated by an acridine orange endothelium bromide (AOEB) staining kit (Jizhi Biochemical Technology, Shanghai), and apoptotic proteins (Caspase-3, Bax, and Bcl-2) were also extracted for western blotting as previously described [22].

### 2.9. Enzyme-Linked Immunosorbent Assay (ELISA)

The culture, seed plate, and grouping of rat NR8383 macrophages (ATCC, SIBS, China) were the same as DRGs. The experimental groups were as follows (*n* = 3): Control group (0.5 mL PBS), Exo-miR-Con group (100 *μ*g pure exosomes in 0.5 mL PBS), and Exo-miR-494 group (100 *μ*g Exo^miR-494^ in 0.5 mL PBS). After 48 hours of coculture, the expression of inflammatory factors was detected by ELISA kits: rat TNF-*α*, IL-6, IL-8, and IL-10 ELISA kit (Thermo Scientific, Shanghai, China).

### 2.10. Establishment of SCI Rat Model and Experimental Grouping

The rats were anesthetized with 4% sodium pentobarbital (50 mg/kg; Beyotime Biotechnology, China). At the T10 level, a 2 cm incision was achieved to expose the spinal skeletal muscle in a sterile environment. Except for the Sham group, the T10 segment was impacted by the spinal cord injury percussion apparatus (Yuyan Instrument, China) in the other groups. The weight of the metal rod reached 25 g, and the height was 50 mm. Subsequently, involuntary twitch and tail swing were identified in both hind limbs of all rats, demonstrating that the injury reached the standard of the spinal cord injury model. In the Sham group, the above operations were performed without spinal cord injury. After suturing the wound, the rats after operation recovered in a warm incubator. In addition, rats in the Sham group underwent the above operations without spinal cord injury.

After operation, the animals were urinated three times a day until they could urinate by themselves. Tail-vein administration was performed per 24 h for 7 consecutive days after establishment of the SCI model. To be specific, the dosage was as follows: Control group and Sham group were administered with 1 mL PBS, while the Exo-miR-con group with 100 *μ*g pure exosomes (diluted in 1 mL PBS), and the Exo-miR-494 group was treated with 100 *μ*g Exo^miR-494^ (diluted in 1 mL PBS).

### 2.11. Exo^miR-494^ Staining

According to one research [23], the dye working solution was prepared. In short, dilute the dye storage solution with diluent C reagent (UR52303, Umibio, China), prepare the PKH67 dye working solution with the concentration of 100 nm, add 10 *μ*L PKH67 dye working solution to the Exo^miR-494^, mix well, and culture at room temperature for 5 min. The reaction between Exo^miR-494^ and PKH67 dye working solution was terminated for 2 min by adding 500 *μ*L 1% bovine serum albumin (BSA). Afterwards, the volume of the mixture was added to 25 mL and then centrifuged for 45 min at 120,000 g. The supernatant was discarded, and the residue was suspended in PBS. The final precipitate was PKH67-Exo^miR-494^.

### 2.12. Drug Toxicity Test of Exo^miR-494^ to the Lung and Liver

After 24 hours of treatment with Exo^miR-494^, the lung and liver tissues of rats in the Exo-miR-494 group were taken out for HE staining and compared with those of healthy rats. Simultaneously, the activities of lactate dehydrogenase (LDH), superoxide dismutase (SOD), and alanine aminotransferase (ALT) in the healthy rat liver were measured by the enzyme labeling method. Firstly, we extract 20 *μ*g liver homogenate, and PBS were used to lyse cells. Then, three enzyme assay kits (Wanlei Bio, China) were added to the cell culture medium, the LDH activity, ALT activity, and SOD activity according to the instructions (Supplement 4, 5, 6). The following are the specific detection methods.

#### 2.12.1. LDH Detection (Visible Light Colorimetry)

First, take 10-50 *μ*L rat liver homogenate. Then, add the LDH kit, mix well, and place in a water bath at 37°C for 15 min; finally, set the wavelength to 440 nm and the optical path to 1 cm and measure the absorbance OD value of each tube with an ultraviolet spectrophotometer (Shimadzu, Japan).

#### 2.12.2. Calculation Formula of Tissue LDH Activity

1.5% is produced in the reaction system when each gram of tissue protein reacts with the matrix at 37°C for 15 min; 1 *μ*mol pyruvate is 1 unit (U).

#### 2.12.3. SOD Detection (Hydroxylamine Method)

First, take 20 *μ*L rat liver homogenate. Then, add the SOD kit, mix well, and place at room temperature for 10 min; finally, set the wavelength to 550 nm and the optical path to 1 cm and measure the absorbance OD value of each tube with an ultraviolet spectrophotometer.

#### 2.12.4. Calculation of Total SOD Activity in Tissue Homogenate

When the inhibition rate of SOD per mg tissue protein in 1 mL reaction solution reaches 50%, the corresponding SOD amount is one SOD activity unit (U).

#### 2.12.5. ALT/GPT Detection (Visible Light Colorimetry)

Mix the ALT kit with 50 *μ*L rat liver tissue; place it at room temperature for 5 min, wavelength 505 nm, optical path 1 cm, and zero with double distilled water; and measure the absorbance OD value of each tube. Check the standard curve to obtain the corresponding ALT/GPT activity unit.

Moreover, after 48 hours of administration, miR-494 were extracted for further quantitative RT-PCR analysis.

### 2.13. Total RNA Isolation and Quantitative Real-Time Polymerase Chain Reaction (qRT-PCR)

RNA extraction kits (Solarbio, China) were adopted to extract total RNA from cells and spinal cord lesion tissues. To be specific, miRNA extraction kits (Solarbio, China) were used for miR-494 in exosomes or cells, target mRNA or miR-494 acted as templates, and reverse transcription kits (Solarbio, China) were employed for reverse transcription into cDNA. Relative quantification of RNA expression levels was performed on an ABI 7500 Real-Time PCR system (Applied Biosystems, Carlsbad, CA). Furthermore, the 2^-∆∆Ct^ method was employed for relative quantification, and U6 was the internal reference gene (refer to Supplement 3 for specific methods).  Table 2 lists all the primers applied in the experiment.

### 2.14. WB Analysis

RIPA buffer was employed to extract the total exosomes, protein of cells, and spinal cord tissue, and WB experiments were performed by complying with the manufacturer's protocol. Primary antibodies include CD63, CD9, GAPDH, *β*-actin, Caspase-3, Bax, Bcl-2, and GAP-43 (rabbit, Abcam, UK).

### 2.15. HE Staining of Spinal Cord Tissue

After 4 weeks of administration, rats in each group were anesthetized with 4% sodium pentobarbital (50 mg/kg). Then, 1 cm spinal cord segments (including injury center) were stained with HE staining. The center of the lesion was the most severe transverse section.

### 2.16. Immunofluorescence Analysis

After 4 weeks of administration, the longitudinal spinal cords of rats in each group were cut into frozen sections at 0.02 mm intervals. Subsequently, the sections were permeabilized in 0.5% Tween solution and blocked with 5% bovine serum, combined with mouse neurofilament (NF-H) antibody (1 : 1000, Abcam, UK) and rabbit glial fibrillary acidic protein (GFAP) antibody (1 : 100, Abcam, UK) overnight at 4°C. Afterwards, the spinal cord sections were incubated with DAPI (1 : 1000, Invitrogen) and goat anti-rabbit IgG (H+L) secondary antibody (1 : 300, Invitrogen) for 1 h, and the image was captured under a confocal fluorescence microscope (Leica Microsystems, Germany).

Furthermore, we use ImageJ software (V1.8.0.112) to measure the relative expression of NF and GFAP by average fluorescence intensity. First, we use the ImageJ software to open the picture of single fluorescence (NF or GFAP), then adjust the threshold (software default), select the appropriate threshold algorithm, and detect and analyze the result. The average fluorescence intensity formula is as follows: average fluorescence intensity = total fluorescence intensity of the area/the area.

### 2.17. Locomotor Capacity Assessment

The locomotor capacity of hind limbs of all rats was assessed by Basso, Beattie, and Bresnahan (BBB) locomotor rating scale. 0 means no motor activity, and 21 means normal motor activity. The mentioned results were observed by two independent researchers at 1 day before and 1, 7, 14, 21, and 28 days after injury.

### 2.18. Paw Withdraw Thermal Latency (PWTL)

On days 0, 7, 14, 21, and 28 after injury, the thermal sensitivity of rats was detected by PWTL. The radiant heat source (Techman Company, China) was placed in the middle of the posterior surface of the rat sole. After thermal stimulation, the rats' hind paw moved or 20 s later, the thermal stimulator would automatically close to avoid tissue damage, and the pain threshold time was recorded. The effects of 50% radiant heat intensity and 6-8 s heat block latency on the normal pain threshold were observed.

### 2.19. Hind Leg Neurophysiological Experiment

After 1 month of treatment, rats in each group underwent the experiment. In brief, 4% sodium pentobarbital (50 mg/kg) were adopted to anesthetize animals. Transcranial electrical stimulation was applied with pulse, and a SEN-7301 constant current isolation stimulator (Nihon Kohden Corp., Japan) was employed for 1 ms at 9 mV. Two 30 g stainless steel stimulation electrodes (Nihon Kohden Corp., Japan) were implanted subcutaneously into the hind limbs of rats to record motor evoked potentials (MEPs) of rat hind limbs until we obtained 3~5 stable and repeatable potentials, and we continuously recorded the amplitude of MEP for 3 times.

### 2.20. Statistical Analysis

SPSS 21.0 statistical software was used (IBM Corp., Armonk, NY, USA). Data were compared by performing two-tailed Student's *t*-test or one-way analysis of variance and Tukey's post hoc test. *p* < 0.05 was considered to be statistically significant.

## 3. Results

### 3.1. Characterization of Exo and Exo^miR-494^

**Figure 2:**
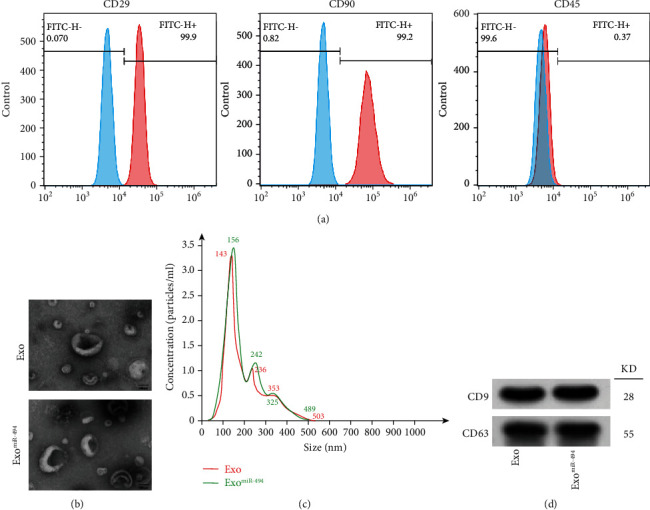
Characterization of BMSC-Exo and Exo^miR-494^. (a) Expression of BMSC markers (CD29, CD45, and CD90) was identified by flow cytometry. (b) Morphological observation of Exo and Exo^miR-494^ by transmission electron microscopy (indicated by yellow arrow). Scale bar = 100 nm. (c) Nanoparticle tracking analysis of Exo and Exo^miR-494^. (d) The BMSC-Exo marker proteins (CD9, CD63) were analyzed using western blot analysis.

As mentioned above, we successfully isolated MSCs. Flow cytometry showed that the isolated BMSCs highly expressed CD29 (99.9%), CD90 (99.2%), and low expressed CD45 (0.37%), which were in accordance with the characteristics of rat BMSCs (Figure 2(a)). Next, we extracted considerable Exo from the supernatant of rat bone marrow MSCs by the kit method and compared Exo with the subsequent construction of Exo^miR-494^. First, we observed Exo/Exo^miR-494^ under TEM by negative staining, and TEM results showed that many Exos and Exo^miR-494^ (the yellow arrow indicates Exos or Exo^miR-494^) had a typical saucer-like structure with a size of approximately 30–200 nm (Figure 2(b)), and NTA also showed that the diameters of Exo and Exo^miR-494^ were concentrated at about 150 nm (Figure 2(c)), which showed that the incorporation of miR-494 did not affect the diameter or morphology of Exo. Moreover, the WB band also showed that miR-494 did not affect the expression of Exo-labeled proteins CD9 and CD63 (Figure 2(d)).

### 3.2. Exosome Loading miR-494 Has Good Loading Rate and Stability

**Figure 3:**
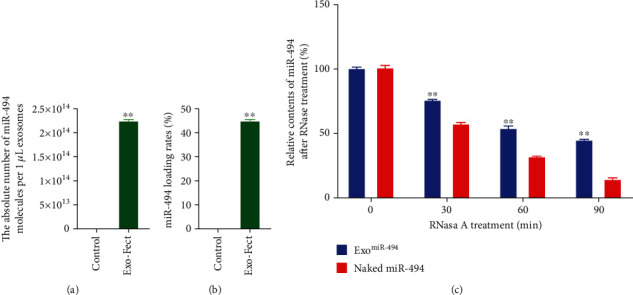
Exosome loading miR-494 by Exo-Fect. (a) The absolute number of miR-494 molecules in exosomes by absolute quantitative real-time PCR. *n* = 3, data are represented as mean ± SD, ^∗∗^*p* < 0.01 vs. Control. (b) The loading rates of miR-494 in Exo were presented. *n* = 3, data are represented as mean ± SD, ^∗∗^*p* < 0.01 vs. Control. (c) Relative contents of naked miR-494 and Exo^miR-494^ after RNase A treatment. *n* = 6, data are represented as mean ± SD, ^∗^*p* < 0.05 and ^∗∗^*p* < 0.01 vs. naked miR-494.

According to existing studies [21], we loaded miR-494 into exosomes by Exo-Fect. The results of absolute quantitative RT-PCR showed that the exosomes of the Exo-Fect group contained more miR-494 molecules, with the number of about 2.26∗10^14^/*μ*g exosomes (Figure 3(a)), and the sample loading rate/transfection efficiency of miR-494 is about 38.24% (Figure 3(b)). Interestingly, the number of molecules in the Control group (untransfected) also had 1.12∗10^11^/*μ*g exosomes, which may be due to the fact that exosomes can also actively take up some miRNAs in very small amounts (Figure 3(b)).

Furthermore, in order to test the stability of Exo^miR-494^ in the circulatory system, we treated naked miR-494 and Exo^miR-494^ with RNase A. The results of RT-PCR showed that the naked miR-494 was degraded gradually with time, and the residual amount of miR-494 was less than 15% at 90 min, while the residual amount of miR-494 in Exo^miR-494^ was still 44.8 ± 2.1% (Figure 3(c)). These results showed that exosomes had a good drug loading effect and could effectively prevent the degradation of miRNAs in body fluid.

### 3.3. Therapeutic Effect of Exo^miR-494^ on DRGs In Vitro

**Figure 4:**
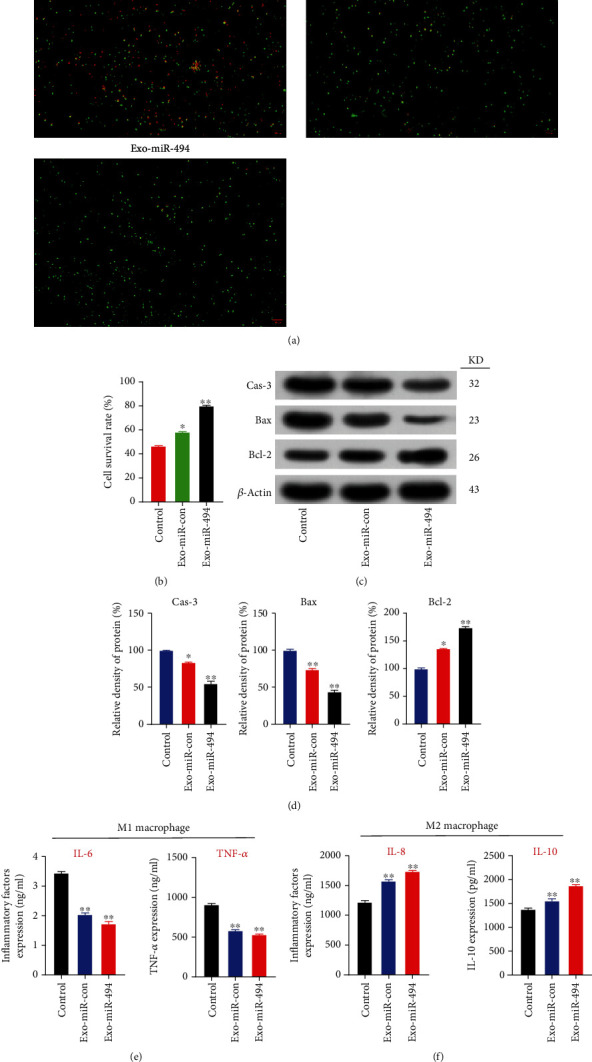
Therapeutic effect of Exo^miR-494^ on DRGs and NR8383 macrophages in vitro. (a) Cell survival of DRGs was measured by AOEB staining in Control, Exo-miR-con, and Exo-miR-494 groups (the living cells are green and the dead cells are red). (b) Cell viability by cell counting in the Control, Exo-miR-con, and Exo-miR-494 groups. *n* = 3, data are represented as mean ± SD, ^∗^*p* < 0.05 and ^∗∗^*p* < 0.01 vs. Control. (c) Western blot analysis of apoptosis-related proteins (Caspase-3, Bax, and Bcl-2) at 24 h in the Control group, Exo-miR-con group, and Exo-miR-494 group. (d) Relative density of Caspase-3, Bax, and Bcl-2. *n* = 6, data are represented as mean ± SD, ^∗∗^*p* < 0.01 vs. Control. (e) ELISA analysis for secretion of M1 polarization-related proinflammatory cytokines (IL-6, TNF-*α*) and (f) M2 polarization-related proinflammatory cytokines (IL-8, IL-10) in LPS/hypoxia-treated NR8383 macrophages at 48 h after ExomiR-494, ExomiR-con, and PBS administrations. *n* = 3, data are represented as mean ± SD, ^∗∗^*p* < 0.01 vs. Control.

Existing studies showed that MSC-derived pure exosomes or pure miR-494 could inhibit neuronal apoptosis [18]. After LPS/hypoxia treatment, the DRGs were treated with PBS, Exo^miR-con^, and Exo^miR-494^. Live/dead cell double staining showed that the DRG survival rate of the Control group was significantly decreased, only 46.35%, while the survival rate of the Exo-miR-494 group was as high as 80.1 ± 2.5% (Figures 4(a) and 4(b)); In addition, the Exo-miR-con group also had a survival rate of 58.2 ± 2.6%, which was attributed to the antiapoptotic effect of the exosome alone (Figures 4(a) and 4(b)). The WB trend of apoptotic protein was the same as the cell survival rate (Figure 4(c)). The Caspase-3 and Bax in the Exo-miR-con group and the Exo-miR-494 group were significantly lower than those in the Control group (Figure 4(d)). On the contrary, the antiapoptotic protein Bcl-2 in the Exo-miR-con and Exo-miR-494 groups was significantly higher than that in the Control group (Figure 4(d)), because both exosome and miR-494 have antiapoptotic effects, which may be coordinated with each other and conform to the cell survival trend.

### 3.4. Exo^miR-494^ Can Promote the Polarity Transformation of Macrophages In Vitro and Inhibit Inflammatory Reaction

MSC-derived exosomes can regulate the polarization of macrophages and reduce the inflammatory response after SCI [24, 25]. Next, we studied the relationship between Exo^miR-494^ and inflammatory response. The alveolar macrophages of NR8383 rats were treated with LPS/hypoxia for 24 h and then cocultured with miR-494, Exo^miR-con^, and Exo^miR-494^ for 48 h. The expression of MI and M2 markers was detected by ELISA. As revealed from the results, Exo^miR-con^ or Exo^miR-494^ treatment significantly decreased the expression of M1 polarization-related proinflammatory cytokines (TNF-6 and IL-*α*), but the difference between the two groups was not obvious (Figure 4(e)). Meanwhile, Exo^miR-con^ or Exo^miR-494^ treatment also significantly increased the expression of M2 polarization-related anti-inflammatory cytokines (IL-8 and IL-10) in Figure 4(f).

### 3.5. Exo^miR-494^ Can Home into the Spinal Cord Lesion in Rats by Tail Vein Injection

**Figure 5:**
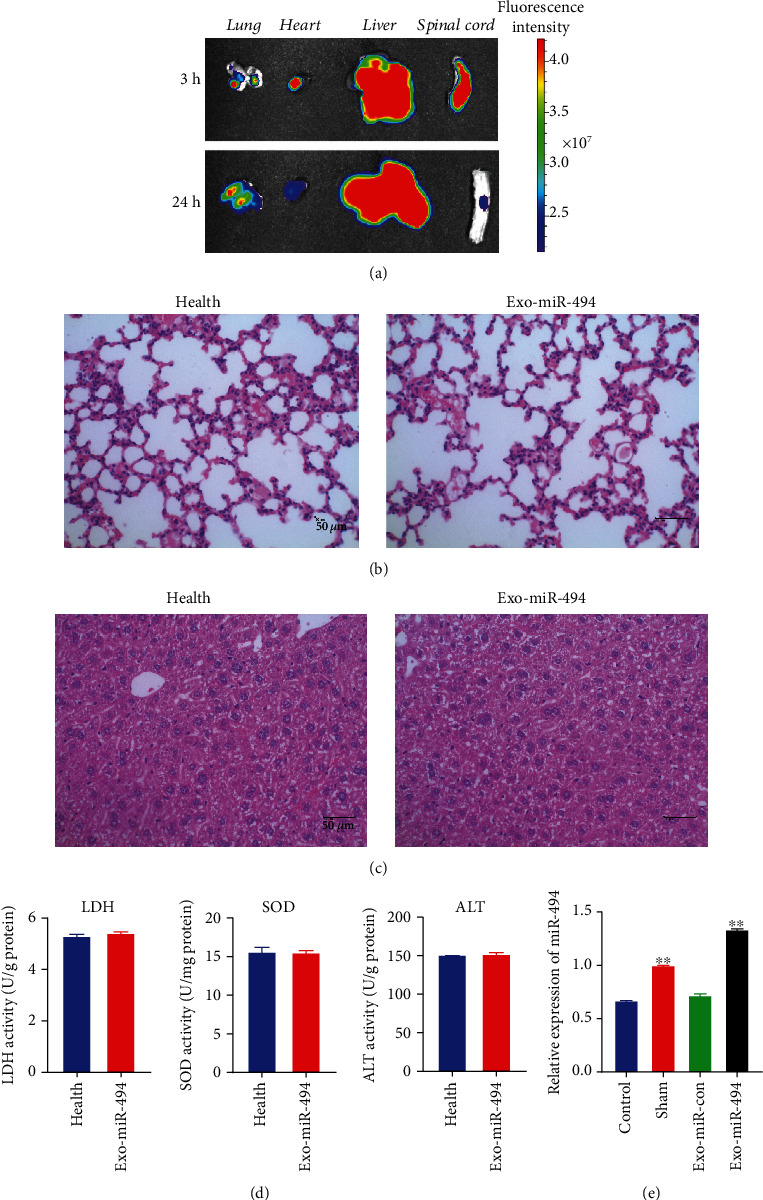
Therapeutic effect of Exo^miR-494^ on spinal cord injury in vivo. Rats were classified into Control group, Sham group, Exo-miR-con group, and Exo-miR-494 group. (a) Fluorescence imaging of organs of Exo-miR-494 group rats at 3 h and 24 h after intravenous injection was illustrated. (b) HE staining of lung in the health group and Exo-miR-494 group. The rats in the health group were fed normally without any injury or treatment. Scale bar = 50 *μ*m. (c) HE staining of liver in health group and Exo-miR-494 group. Scale bar = 50 *μ*m. (d) Enzyme activities of LDH, SOD, and ALT in the liver of health group and Exo-miR-494 group rats. *n* = 6. (e) The relative expressions of miR-494 in injured spinal cord at 48 h after Exo^miR-494-con^, Exo^miR-494^, and PBS treatment by RT-PCR. *n* = 6, data are represented as mean ± SD, ^∗∗^*p* < 0.01 vs. Control.

Next, animal experiments were performed. The DIR iodide-labeled Exo^miR-494^ were injected into SCI rats via the tail vein, and its distribution in the organs and spinal cord was observed under the animal fluorescence imaging system. As revealed from the results, 3 h imaging results showed that Exo^miR-494^ showed relatively high fluorescence intensity in the lung and liver, which was consistent with existing research results (Figure 5(a)) [26], that is, exosomes injected into the body were mainly phagocytized by mononuclear in the liver. The results of 24 h showed that the fluorescence intensity of all Exo^miR-494^ decreased significantly, demonstrating that Exo^miR-494^ were absorbed by liver cells and nerve cells in the injured area (Figure 5(a)).

### 3.6. Exo^miR-494^ Injected by Tail Vein Has No Toxic Effect on the Liver

According to the fluorescence images of animals (Figure 5(a)), we know that most of Exo^miR-494^ converges to the lung and liver, so we conducted a toxicity test on healthy rats after administration of Exo^miR-494^. The HE staining results showed that the lung and liver of the Exo-miR-494 group showed a normal shape and contour, without obvious signs of injury (Figures 5(b) and 5(c)). Furthermore, the activities of enzymes closely related to liver function were detected. The results demonstrated that the activities of LDH, SOD, and ALT in rat liver after Exo^miR-494^ treatment were similar to those in healthy rats, indicating that the exosome has no toxic side effects on rat liver, which is an ideal miRNA vector (Figure 5(d)).

### 3.7. Exo^miR-494^ Can Reduce the Lesion Volume of the Spinal Cord

**Figure 6:**
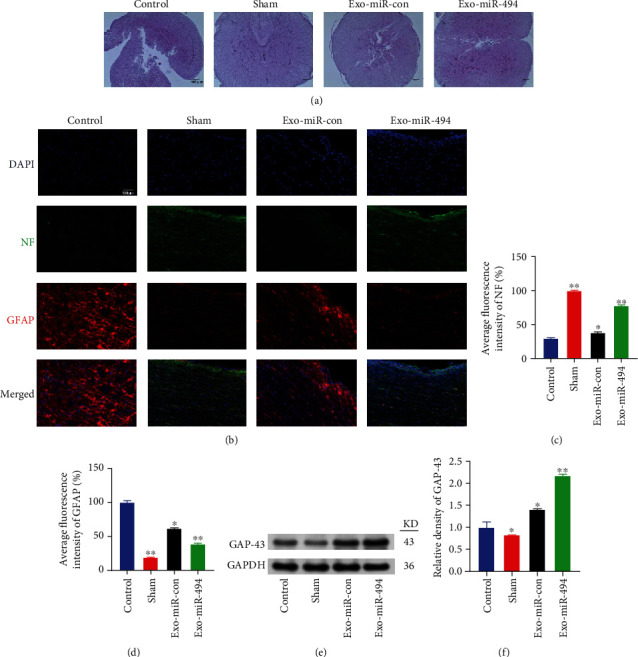
Exo^miR-494^ can reduce the lesion volume of spinal cord and promote the regeneration of neurofilament. (a) On the 28th day after injury, the transverse section of spinal cord in each group was stained with HE. Scale bar = 100 *μ*m. (b) Immunofluorescence images showing the expressions of neurofilament (NF, green) and astrogliosis (GFAP, red) in longitudinal section of the spinal cord lesion at 4 weeks in each group. Scale bar = 200 *μ*m or 50 *μ*m. (c) Relative average fluorescence intensity of NF (%) in each group rats by ImageJ software. The average fluorescence intensity in Sham group was set to 100%. *n* = 6, data are represented as mean ± SD, ^∗^*p* < 0.05 and ^∗∗^*p* < 0.01 vs. Control. (d) Relative average fluorescence intensity of GFAP (%) in each group of rats. The average fluorescence intensity in Control group was set to 100%. *n* = 6, data are represented as mean ± SD, ^∗^*p* < 0.05 and ^∗∗^*p* < 0.01 vs. Control. (e) Expression of GAP-43 in rats of each group after 28 days of SCI. (f) Relative density of GAP-43 by ImageJ software. *n* = 6, data are represented as mean ± SD, ^∗^*p* < 0.05 and ^∗∗^*p* < 0.01 vs. Control.

Next, we evaluated the injury volume of the transverse section of the spinal cord by HE staining. At day 28 post-SCI, the lesion center of the Control group often contained typical vacuoles with irregular shape (Figure 6(a)). Compared with the Control group, the central contour of Exo-miR-494 was more complete, the shape was more normal, and the damage volume was smaller (Figure 6(a)). In addition, the damage degree of the Exo-miR-con group was between that of the Control group and the Exo-miR-494 group (Figure 6(a)).

### 3.8. Exo^miR-494^ Promotes the Regeneration of Neurofilament in Spinal Cord Injury Rats

To determine the effect of Exo^miR-494^ on neurofilament regeneration, NF-H and GFAP double-labeled immunofluorescence were employed to evaluate neurofilament regeneration and astrocyte activation 4 weeks after injury and as revealed from the results (Figure 6(b)). As a result, considerable active astrocytes were recruited and the neurofilament disintegrated in the Control group (Figure 6(b)), and the NF-positive cells in the Exo-miR-494 group were significantly higher than those in the Control group and the Exo-miR-con group (Figure 6(c)); also, the GFAP-positive cells were lower than in the Control group and the Exo-miR-con group (Figure 6(d)). Thus, the Exo-miR-494 group rats showed significantly increased neurofilament and decreased astrocytes (Figure 6(d)).

At the same time, after 28 days of injury, we measured the expression of GAP-43 (Figure 6(e)). As a result, after injury, GAP-43 was upregulated in all three groups except the Sham group (Figure 6(f)). Among them, the upregulation was the highest in the Exo-miR-494 group, up to 110 ± 4.0%, and there was also a certain increase in the Exo-miR-con group (Figure 6(f)), which was derived from simple exosomes and promoted the increase of GAP-43.

### 3.9. Exo^miR-494^ Promotes the Recovery of Behavioral Function in Rats with Spinal Cord Injury

**Figure 7:**
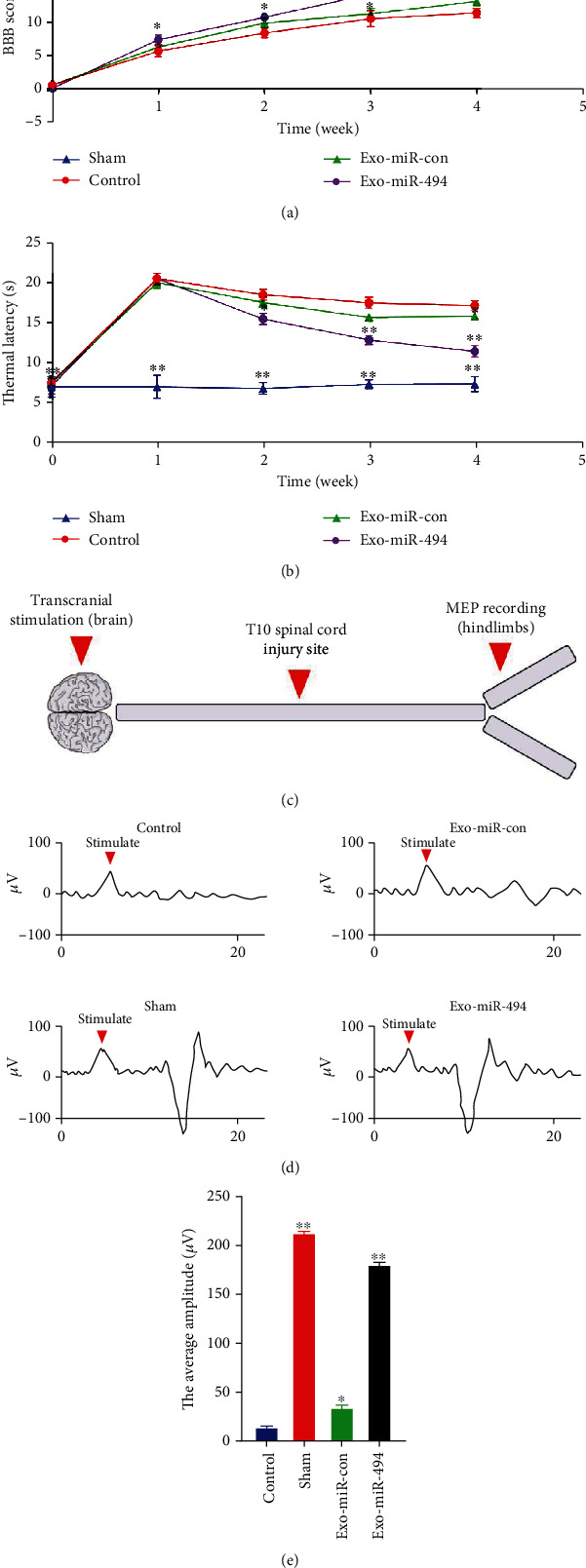
Exo^miR-494^ promotes functional recovery in SCI rats. (a) BBB scores of rats in Control group, Sham group, Exo-miR-con group, and Exo-miR-494 group. *n* = 6, data are represented as mean ± SD, ^∗^*p* < 0.05 and ^∗∗^*p* < 0.01 vs. Control. (b) The PWTL of rats in Sham group, Control group, Exo-miR-con group, and Exo-miR-494 group. *n* = 6, data are represented as mean ± SD, ^∗^*p* < 0.05 and ^∗∗^*p* < 0.01 vs. Control. (c) Schematic diagram of neuroelectrophysiology. (d) MEPs of rats in Sham group, Control group, Exo-miR-con group, and Exo-miR-494 group after 4 weeks of treatment (the average of the three times was collected into a line graph, and the red arrow marked the stimulus artifact). (e) The average amplitude in Sham group, Control group, Exo-miR-con group, and Exo-miR-494 group after 4 weeks of treatment. *n* = 6, data are represented as mean ± SD, ^∗^*p* < 0.05 and ^∗∗^*p* < 0.01 vs. Control.

Lastly, we evaluated the behavioral function of rats. To determine the effect of Exo^miR-494^ on the recovery of the motor function of all rats, the BBB score was used to evaluate the locomotor capacity of all rats at each time point after injury, and as revealed from the results, the BBB scores of rats in each group increased gradually with time due to SCI rats also having a certain self-restoring capacity (Figure 7(a)). On day 28, both Exo-miR-494 group and Exo-miR-con group animals showed significant functional recovery, of which the average score of the Exo-miR-494 group was 15.8 ± 0.7 and that of Exo-miR-con animals was 12.7 ± 0.5 (Figure 7(a)). The average score of the Control group was lower, which was only 11.2 ± 0.65 (Figure 7(a)). So, the motor functional recovery of the Exo-miR-494 group was better than those of the Exo-miR-con group and Control group.

To further analyze thermal sensitivity by PWTL, on the first day before injury, all rats showed almost the same and normal PWTL, with an average of about 6.8 ± 0.5 s (Figure 7(b)). One week after spinal cord injury, PWTL of rats in each group was more than 20 s due to severe trauma (Figure 7(b)). Then, the PWTL of rats in each group decreased gradually, and the most obvious decrease was in the Exo-miR-494 group, reaching 12.7 ± 0.4 s on the 28th day, 16.5 ± 0.8 s in the Exo-miR-con group, and 17.5 ± 0.3 s in rats of the Control group (Figure 7(b)).

### 3.10. Exo^miR-494^ Promotes the Recovery of Neuroelectrophysiology in Spinal Cord Injury Rats

4 weeks after injury, we conducted neuroelectrophysiological experiments (Figure 7(c)). As shown in Figures 7(d) and 7(e), the MEPs in hind limbs of all rats showed that there was an obvious one-way wave in the Exo-miR-494 group, and the average amplitude (184 ± 4 *μ*V) was significantly larger than that in the Exo-miR-con (34 ± 5 *μ*V) or Control groups (14 ± 5 *μ*V). Therefore, the average amplitude of MEP in the Control group was very small, which was due to the ineffective recovery of neurophysiological function.

## 4. Discussion

SCI is capable of causing severe motor and sensory disorders in patients, and no effective treatment has been applied to the clinic thus far [27]. It is increasingly evidenced that many miRNAs are involved in the pathogenesis or recovery mechanism. To be specific, miR-135a-5p can promote the recovery of spinal cord injury by mediating ROCK pathway [28], and low-level miR-130a-3p can activate the IGF-1/IGF-1R pathway to relieve pathological pain after spinal cord injury [29].

miR-494 refers to a tumor suppressor inhibiting the proliferation and colony formation of tumor cells [30]. miR-494 can reduce autophagy and apoptosis of PC-12 cells induced by LPS by targeting IL-13 in vitro [18]. Besides, miR-494 can also regulate the PTEN/Akt/mTOR pathway, thereby inhibiting neuronal apoptosis after SCI in rats [22, 31]. Furthermore, as reported by our previous research, SIRT1 can inhibit apoptosis after spinal cord injury via miR-494 [32].

Stem cell transplantation has been investigated in tissue regeneration for years; however, it still has some limitations [33, 34]. Over the past few years, exosomes, a novel type of intercellular communication device, have been employed as a good biological carrier for local or systemic small RNA delivery for treating stroke or spinal cord injury and other central nervous system diseases. The mentioned exosomes play an active role in tissue regeneration, while acting as the carriers of genetic materials (e.g., mRNAs, miRNAs, and lncRNAs), which can be used as gene transfer systems for alternative cell therapy. Here, Exo^miR-494^ was constructed by chemical transfection, and the therapeutic effect of Exo^miR-494^ on SCI was studied in depth.

First, miR-494 was loaded into the exosome effectively by chemical transfection, and it exhibited high loading efficiency, about 38% loading rate, complying with the existing results [21]. The amount of exosomes secreted in vivo is very small, so convenience was provided for the mass production of Exo^miR-494^ by chemical transfection, which is considered a high loading efficiency method. Besides, exosomes exhibit excellent encapsulation and antidegradation properties, capable of effectively preventing the degradation of target miRNA in vivo environment, which provides the possibility for the tail vein administration of Exo^miR-494^. It is generally known that innate immune cells (i.e., microglia and astrocytes) and infiltrating leukocytes (i.e., macrophages and neutrophils) are activated after SCI, thereby leading to inflammatory cascade. The mentioned inflammatory cells release various neurotoxins, proinflammatory cytokines, chemokines, free radicals, excitotoxic amino acids, nitric oxide, and others, creating a very unfavorable microenvironment for the regeneration of neurons [35]. In addition, glial scar imposes a major obstacle on axonal regeneration. After spinal cord injury, injured fibroblasts invade the injured area and then secrete considerable extracellular matrix (e.g., type IV collagen, fibronectin, and laminin), constituting the main component of the glial scar [35, 36]. Wang et al. reported that miR-494 can inhibit the proliferation and synaptic remodeling of spinal reactive astrocytes in SCI rats by activating the Nogo/NgR signaling pathway [19]. Reactive astrocytes are recognized as the pathological markers of central nervous system damage (e.g., Parkinson's disease, Alzheimer's disease, and spinal cord injury) [37]. Reactive astrocytes are capable of secreting considerable extracellular matrix and forming glial scar, thereby leading to limited nerve repair and axonal degeneration [38, 39]. Moreover, glial scar formation is capable of stimulating the production of GFAP, which activates the RhoA signal, causes the growth cone to collapse, and inhibits axon regeneration [40].

In this study, as revealed from NF and GFAP immunofluorescence results, Exo^miR-494^ can effectively downregulate the expression of GFAP, as well as promote the increase of the neurofilament. Indeed, the mentioned positive results reveal the important role of miR-494 for treating spinal cord injury. Over time, the BBB score of Exo^miR-494^ group was significantly higher than those of other groups, demonstrating that the motor function of rats had been significantly improved. In addition, to explore the effect of Exo^miR-494^ on the electrophysiological function of the hind limbs of SCI rats, the electrophysiological study was conducted on the hind limbs of rats in 4 weeks. Complying with the existing results, Exo^miR-494^ is capable of effectively facilitating the recovery of the neuroelectrophysiological function of SCI rats.

## 5. Conclusion

In brief, the results showed that the exosome is an excellent miRNA vector, the tail vein injection of Exo^miR-494^ can upregulate the expression of miR-494, improve the local immune environment, and inhibit neuronal apoptosis and release of proinflammatory factors, thereby promoting the regeneration of the neurofilament and the recovery of behavioral function.

## Figures and Tables

**Table 1:** Synthesized miR-494 mimics and negative control (NC).

Gene	Sequence (5′ to 3′)
miR-494 mimic	UGAAACAUACACGGGAAACCUC
miR-494 mimic NC	UUCUCCGAACGUGUCACGU

**Table 2:** Primers used for RT-qPCR.

Gene	Sequence (5′ to 3′)
miR-494	Forward CATAGCCCGTGAAACATA CACG Reverse GTGCAGGGTCCGAGGT
U6	Forward CGCTTCGGCAGCACATATACTA Reverse GCGAGCACAGAATTAATACGAC

## Data Availability

The [experimental data] data used to support the findings of this study are included within the article.

## Supplementary Materials

Supplementary 1 Supplement 1: instruction manual of the GS exosome isolation reagent kit.

Supplementary 2 Supplement 2: Exo and Exo-miR-494 negative staining steps for transmission electron microscope.

Supplementary 3 Supplement 3: real-time polymerase chain reaction (RT-PCR) experimental steps.

Supplementary 4 Supplement 4: ALT test kit instructions.

Supplementary 5 Supplement 5: LDH test kit instructions.

Supplementary 6 Supplement 6: SOD test kit instructions.
