# Supplementary material for: Rat Bone Mesenchymal Stem Cell-Derived Exosomes Loaded with miR-494 Promoting Neurofilament Regeneration and Behavioral Function Recovery after Spinal Cord Injury
Source: Oxid Med Cell Longev. 2021 Oct 1;2021:1634917. doi: 10.1155/2021/1634917 (PMC8501401; doi:10.1155/2021/1634917)
Supplement: Supplementary 1 — Supplement 1: instruction manual of the GS exosome isolation reagent kit. [file 1634917.f1.pdf]

GS™ Exosome Isolation Reagent (for serum or cell supernatant)

产品简介

Exosome（外泌体） 是活细胞分泌的直径约为 30~150nm 的膜性囊泡，天然存在于体液中，包括血液、唾液、尿液和母乳等。Exosome 携带了包含细胞溶质蛋白、参与细胞内信号转导的蛋白、各种代谢酶、热休克蛋白和四跨膜蛋白及特殊蛋白质，参与细胞活动的重要调控，有望成为多种疾病的早期诊断标志物。Exosome 在基因和药物运载、肿瘤和肿瘤生物学研究方面已经成为热点。

GS™ Exosome Isolation Reagent 是用于快速高效地从血清血浆、细胞培养上清、脑脊液和尿液中提取 exosome 的试剂盒，仅需通过简单混匀和常规离心即可从样本中获取大量结构完整的 exosome，比传统的超速离心法节省时间和样本。以GS™ Exosome Isolation Reagent 提取的exosome 可用于 Western Blot、ELISA、蛋白质谱、qPCR 等后续实验。

试剂组分

货号	产品名称	规格	抽提体积
E5001	GS™ Exosome Isolation Reagent A 液	50 mL	150 mL
	GS™ Exosome Isolation Reagent B 液		

运输保存

常温运输。请于 4℃保存，可稳定保存一年。

使用方法

1 样品准备

血清：

1) 血清样本室温，2,000×g 离心 10 min，以去除残留细胞及碎片；
2) 收集上清液，进行 exosome 提取或 4℃保存。

血浆：

1) 血浆样本室温，2,000×g 离心 10 min，以去除残留细胞及碎片；
2) 转移上清液至新的离心管中，室温，10,000×g 离心 2 min，收集上清液，进行 exosome 提取或 4℃保存。

注： 若后续实验为提取 RNA，建议使用 500 μL 血清血浆； 若为提取蛋白，建议使用 300 μL 血清血浆。

2 Exosome 提取

1) 转移上清液至新的离心管中，加入 1/3 倍上清体积的 A 液。
2) 颠倒混匀或移液器吹打混匀，4℃放置 15 min。
3) 室温，13,000×g 离心 10 min，小心吸弃上清； 13,000×g 离心 30 s，小心吸弃残留上清。
4) 加入与 Reagent A 等体积的 B 液，使用移液器吹打混匀，13,000×g 离心 10 min，小心吸弃上清。
5) 沉淀即为 exosome，-80℃保存。若提取 RNA 或蛋白，使用 100 ~ 200 μL RNase-free H_2O 重悬，进行后续实验。
