# Supplementary material for: Rat Bone Mesenchymal Stem Cell-Derived Exosomes Loaded with miR-494 Promoting Neurofilament Regeneration and Behavioral Function Recovery after Spinal Cord Injury
Source: Oxid Med Cell Longev. 2021 Oct 1;2021:1634917. doi: 10.1155/2021/1634917 (PMC8501401; doi:10.1155/2021/1634917)
Supplement: Supplementary 2 — Supplement 2: Exo and Exo-miR-494 negative staining steps for transmission electron microscope. [file 1634917.f2.docx]

**Exo and Exo^miR-494^ negative staining steps for transmission electron microscope**

**Exo / Exo^miR-494^ was fixed on the sample carrier**

1. Resuspend Exo / Exo^miR-494^ to 50-100 μ L 2% PFA and mixed with the same amount of 4% PFA;

*Note: Exo / Exo^miR-494^ in 2% PFA can be stored at 4 ℃ for one week.*

2. Will 5 μL Exo / Exo^miR-494^ suspension was added to the Formvar carbon sample carrier; It can also be changed from 5 to 10 μ L of Exo / Exo^miR-494^ suspension was added to the sealing film, and the copper mesh was placed on the suspension with the film face down. Prepare 2-3 copper meshes for each sample;

3. Will be 100 μL PBS was added to the sealing film. The copper mesh (Formvar film face down) was placed on the PBS droplet for cleaning with tweezers;

Important: in all steps, keep the film surface moist and the other side dry.

*Note: the copper mesh of the same sample can be cleaned in the same PBS droplet.*

4. Put the copper net at 50 μL 1% glutaraldehyde for 5 min;

5. Put the copper mesh in 100 μL dd H_2_O 2 min (wash 8 times);

**Negative staining and electron microscopic examination of Exo / Exo^miR-494^**

1. Put the copper net at 50 μL uranyl oxalate solution with pH value of 75 min;

2. The copper mesh was placed on 50 methylcellulose drops for 10 min and operated on ice;

3. The copper mesh is placed on the stainless steel ring at the top of the sample table to absorb the excess liquid on the filter paper;

4. Drying in air for 5 to 10 min;

5. The copper mesh was placed in a box and the electron microscope was taken at 80 kV.
