# Supplementary material for: Rat Bone Mesenchymal Stem Cell-Derived Exosomes Loaded with miR-494 Promoting Neurofilament Regeneration and Behavioral Function Recovery after Spinal Cord Injury
Source: Oxid Med Cell Longev. 2021 Oct 1;2021:1634917. doi: 10.1155/2021/1634917 (PMC8501401; doi:10.1155/2021/1634917)
Supplement: Supplementary 3 — Supplement 3: real-time polymerase chain reaction (RT-PCR) experimental steps. [file 1634917.f3.docx]

**Real-Time Polymerase Chain Reaction (RT-PCR) experimental steps**

1. **Absolute quantitative Real-Time Polymerase Chain Reaction (A-qRT-PCR) to detect the molecular number of Exo^miR-494^.**

Firstly, primers of miR-494 were designed and synthesized (Table 1).

**Table 1. Primers used for RT-qPCR**

| Gene | Sequence (5' to 3') |
| --- | --- |
| miR-494 | Forward CATAGCCCGTGAAACATA CACG  Reverse GTGCAGGGTCCGAGGT |
| U6 | Forward CGCTTCGGCAGCACATATACTA  Reverse GCGAGCACAGAATTAATACGAC |

After the Exo loading test by chemical transfection, the solution after chemical transfection reaction was taken out, transferred to a 1.5ml 100kd ultrafiltration concentration tube, filled with buffer solution to 500uL, concentrated by 8000g / min ultrafiltration, washed to remove the miR-494 outside the exosome, and recovered the exosome. Next, miR-494 in exosomes were extracted with miRNA Extraction Kit (Sangon Biotech Co，Shanghai，China) and eluted with 25ul water. Finally, RT primers were used for SYBR Green I real-time quantitative PCR (absolute quantitative) to analyze the miR-494 expression.

**Table 2. Reverse transcription reaction system**

| Component | volume |
| --- | --- |
| RNA | 10μL |
| Cervical loop primer（20uM） | 2μL |
| Heat shock at 75 ℃ for 3 min, precooling on ice |  |
| 5 ×Reaction Mix | 4μL |
| RRI Enzyme | 1μL |
| M-MLV | 1μL |
| dNTP（10mM） | 2uL |
| Incubation at 42 ℃ for 60 min | |
| It was inactivated by heating at 85 ℃ for 5 s and stored at - 20 ℃ | |

The cDNA of the sample was diluted from 20μL to 100μL, and 2μL / reaction was taken for detection.

20 μL optimal reaction system was as follows,， volume

2×SYBR Premix EX-Taq Mix 10 μL

QF (10 μmol/L) 0.5 μL

QR (10 μmol/L) 0.5 μL

RNase Free dH_2_O 7 μL

Template cDNA 2 μL

Six concentration gradients were made for the standard sample, and three repeat wells were made for each gradient; Make 3 holes for each test sample. Each reaction standard and sample to be tested were added with 2 μL template, so the concentration range of the standard is 9.03 × 10^11^ copies ~ 9.03 × 10^6^ copies / reaction.

The optimal reaction conditions are as follows,

94 ℃ 30 s 1 cycle

94 ℃ 5 s

PCR 40 cycles

61 ℃ 50 s

97℃ 10 s

Melting curve 65℃ 60 s 1 cycle

97℃ 1 s

Finally, the copy number of each sample was calculated according to the standard curve, and the miR-494 absolute copy number of each sample was obtained by combining with its DNA concentration or volume.

1. **Quantitative Real-Time Polymerase Chain Reaction (qRT-PCR) to detect the relative content of miR-494 after RNase treatment (compared with naked miR-494)**

（1） Total RNA extraction

The extraction miR-494 method is described above,

（2） The reverse transcription reaction system is as follows,

DEPC water 9μL

dig primer 1μL

five × buffer 4μL

10M dNTPmix 2μL

RNase inhibitor 1μL

Total RNA 2ul 70 ℃ 5min

Reverse transcriptase 1μL

20ul 42ºC 60min

70ºC 10min

4ºC ∞

（3） The PCR reaction system was as follows

DEPC water (high pressure double steam water) 17.5μL

ten × Taq buffer 2.5μL

MgCl2 2.0μL

10M dNTP Mix 0.5μL

The upstream primer was 0.5μL

The downstream primer was 0.5μL

Tap enzyme (5u / UL) 0.5μL

The total cDNA was 1.0μL

25μL

Parameter setting of PCR instrument

94 ºC

5min 94 ºC -ºC 72 ºC

30s 45s 45s 72ºC 4ºC

7min ∞

1. The relative content of 494 was calculated.
2. **qRT-PCR to detect the content of miR-494 in spinal cord of SCI rats.**

miR-494 of SCI rats were extracted with miRNA extraction kit, and the relative content of miR-494 of SCI rats was calculated according to “2.Quantitative Real-Time Polymerase Chain Reaction (qRT-PCR) to detect the relative content of miR-494 after RNase treatment (compared with naked miR-494)” in the second part.
