# Supplementary material for: Rat Bone Mesenchymal Stem Cell-Derived Exosomes Loaded with miR-494 Promoting Neurofilament Regeneration and Behavioral Function Recovery after Spinal Cord Injury
Source: Oxid Med Cell Longev. 2021 Oct 1;2021:1634917. doi: 10.1155/2021/1634917 (PMC8501401; doi:10.1155/2021/1634917)
Supplement: Supplementary 4 — Supplement 4: ALT test kit instructions. [file 1634917.f4.pdf]

谷丙转氨酶(ALT/GPT)试剂盒(赖氏法)可见光比色法

100管/50样 **WLA113**

仅用于科学研究,不能用于诊断

产品信息

产品名称 谷丙转氨酶（ALT/GPT）试剂盒（赖氏法）可见光比色法

产品概述 谷丙转氨酶（ALT）在37℃及PH7.4条件下，作用于丙氨酸及α-酮戊二酸组成的底物，生成丙酮酸及谷氨酸。反应30min后（固定时间）加入2,4-二硝基苯肼（DNPH）盐酸溶液，既中止反应，同时DNPH与酮酸中羰基加成，生成丙酮酸苯腙。苯腙在碱性条件下呈红棕色，于505nm比读吸光度并计算酶活力。

包装信息

试剂名称	WLA113 (100管/50样)	保存条件
基质缓冲液	50ml	4℃，避光
2，4-二硝基苯肼	50ml	4℃，避光
4mol/L NaOH 溶液	50ml	室温密封
2mmol/L丙酮酸钠标准液	1支	4℃
0.1mol/L磷酸盐缓冲液	1支	4℃

保存日期 本试剂盒自订购之日起6个月内有效。

操作流程

1. 试剂配制：

0.4mol/L NaOH 溶液的配制：临用时按4mol/L NaOH 溶液：双蒸水=1：9 的比例稀释，需多少配多少，室温密封保存。

2. **操作表：**

试剂名称	测定管	对照管
待测样本（ml）	0.1	
基质液（ml）37℃已预温5min	0.5	0.5
混匀后，37℃水浴30min		
2,4-二硝基苯肼（ml）	0.5	0.5
待测样本（ml）		0.1
混匀后，37℃水浴20min		
0.4mol/L NaOH（ml）	5	5

混匀，室温放置5min，波长505nm，光径1cm，双蒸水调零，测定各管吸光度OD值，以（绝对OD值=测定管OD值-对照管OD值），查标准曲线，求得相应的ALT/GPT活力单位。

注意事项

1. 赖氏法标准曲线所定单位数，是用实验方法和卡门氏分光光度法（速率法）作对比测定求得的。以卡门氏单位报告结果，比较准确。**卡门氏单位定义为**：1ml血清，反应液总容量3ml，波长340nm，1cm光径，25℃，1min内所生成的丙酮酸，使NADH氧化成NAD^+而引起吸光度每下降0.001为一个单位（1卡门氏单位=0.482 IU/L，25℃）。
2. 一般血清标本内源性丙酮酸很少，个体相差也不大,作大批标本测定时，不需每份标本都作对照管,严重脂血、黄疸、溶血及陈旧血清须作自身对照管。
3. 酶活力超过150单位时，用盐水稀释血清后重测。
4. 应将一般血清的对照管（或称标本空白管）的吸光度作为日常质控的指标之一；如相差大，可考虑α-酮戊二酸浓度、DNPH浓度及仪器等原因引起。
5. 血清中ALT在室温（25℃）可保存2天，在4℃可保存一周，在-25℃可保存1个月。

谷丙转氨酶(ALT/GPT)试剂盒(赖氏法)可见光比色法

100管/50样 **WLA113**

仅用于科学研究,不能用于诊断

产品信息

组织GPT活力计算公式：

组织中GPT活性（U/gprot）= 通过标准曲线得匀浆液GPT活力（U/L）÷ 匀浆蛋白浓度（gprot/L）

附标准曲线制备：

管号	0	1	2	3	4	5
0.1mol/L磷酸盐缓冲液（ml）	0.10	0.10	0.10	0.10	0.10	0.10
2mmol/L丙酮酸钠标准液（ml）	0	0.05	0.10	0.15	0.20	0.25
基质缓冲液（ml)	0.50	0.45	0.40	0.35	0.30	0.25
2,4-二硝基苯肼（ml）	0.50	0.50	0.50	0.50	0.50	0.50

混匀后，37℃水浴20min

0.4mol/L NaOH 溶液（ml）	5	5	5	5	5	5

混匀，室温放置10min，波长505nm，光径1cm，双蒸水调零，测定各管吸光度值。

各管吸光度减去零管吸光度,所得差值为横坐标,相应的卡门氏单位为纵坐标，绘制标准曲线。

相当于酶活力卡门氏单位	0	28	57	97	150	200
绝对吸光度参考值	0	0.097	0.185	0.276	0.364	0.435

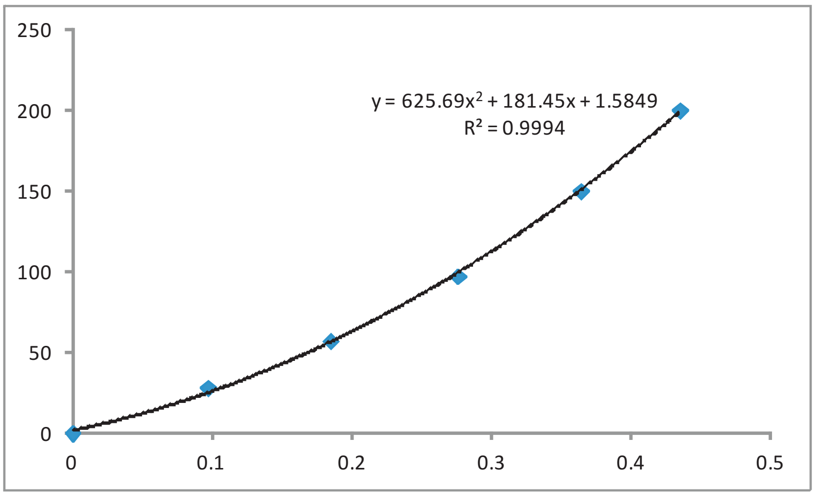

附肝脏样本前处理：

准确称取组织重量，按重量（g）：体积=1：9 的比例，加入9倍体积的生理盐水，冰水条件下机械匀浆，制备成10%的组织匀浆，2500转/分，离心10分钟，取上清液，再用生理盐水10倍稀释成1%的浓度待测。（同时取部分上清液测蛋白浓度，蛋白定量试剂盒本公司有售，货号为WLA004）
