# Supplementary material for: Rat Bone Mesenchymal Stem Cell-Derived Exosomes Loaded with miR-494 Promoting Neurofilament Regeneration and Behavioral Function Recovery after Spinal Cord Injury
Source: Oxid Med Cell Longev. 2021 Oct 1;2021:1634917. doi: 10.1155/2021/1634917 (PMC8501401; doi:10.1155/2021/1634917)
Supplement: Supplementary 5 — Supplement 5: LDH test kit instructions. [file 1634917.f5.pdf]

乳酸脱氢酶（LDH）检测试剂盒（可见光比色法法）

24T **WLA073a** 48T **WLA073b**

仅用于科学研究,不能用于诊断

产品信息

产品名称

乳酸脱氢酶（LDH）检测试剂盒（可见光比色法）

产品概述

乳酸脱氢酶（lactate dehydrogenase，LDH）是一种稳定的蛋白质，存在于正常细胞的胞质中，一旦细胞膜受损，LDH即被释放到细胞外，通过检测细胞培养上清中LDH活性，可判断细胞受损程度，在一些应用中，比如药物筛选时，需要进行特定物质细胞毒性检测。LDH能催化乳酸生成丙酮酸，丙酮酸与2，4-二硝基苯肼反应生成丙酮酸2，4-二硝基苯腙，在碱性溶液中呈棕红色，通过比色可求出酶活力。

本试剂盒可测各种组织、血清（浆）及培养细胞、培养上清液、脑脊液等样本中LDH活性。

包装信息

试剂名称	WLA073a (24T)	WLA073b (48T)	保存条件
基质缓冲液	30ml	60ml	4℃
辅酶 I	粉剂x1	粉剂x1	-20℃
2，4-二硝基苯肼	30ml	60ml	4℃，避光
4mol/L NaOH 溶液	30ml	60ml	4℃
2mmol/L丙酮酸钠标准液	1ml	1ml	4℃

保存日期

本试剂盒自粉剂溶解之日起3月内有效。

操作流程

1. 试剂配制：

（1）辅酶 I 应用液的配制：每支粉剂加1.3ml双蒸水溶解，溶解后即为10x辅酶 I 储备液，如需多次使用建议分装冷冻，防止反复冻融。测定时将10x辅酶 I 储备液用双蒸水10倍稀释，用多少配多少，现用现配。

（2）0.4mol/L NaOH 溶液配制：将4mol/L NaOH 溶液用双蒸水10倍稀释，用多少配多少，现用现配。

2. **操作表：**

试剂名称	空白孔	标准孔	测定孔	对照孔
双蒸水（ml）	0.05+a	0.05		0.05
2mmol/L 标准液（ml）		a		
待测样本（ml）			a	a
基质缓冲液（ml）	0.25	0.25	0.25	0.25
辅酶 I 应用液（ml）			0.05	
充分混匀，37℃水浴，15min				
2，4-二硝基苯肼（ml）	0.25	0.25	0.25	0.25
充分混匀，37℃水浴，15min				
0.4mol/L NaOH 溶液（ml）	2.5	2.5	2.5	2.5

混匀，室温放置 3 min，波长440nm，光径1cm，双蒸水调零，测定各管吸光度值。

注（1）参考取样量：0.2%小鼠脑组织匀浆取10-50μl，大鼠血清取10-30μl。若样本中LDH酶活力太大，可将样本用生理盐水稀释后再测。

注（2）测定空白管中不加辅酶 I 应用液。

注（3）严格按照说明书操作，不可先加辅酶 I 再加基质液。

产品信息

3. **计算公式：**

（1）血清（浆）LDH计算公式：

单位定义：1000ml 血清（浆）37℃与基质作用15min，在反应体系中产生1μmol丙酮酸为1单位。

$$\text{血清（浆）中LDH 活性（U/L）} = \frac{\text{测定OD值-对照OD值}}{\text{标准OD值-空白OD值}} \times \text{标准品浓度（2mmol/L）} \times \text{样本测定前稀释倍数} \times 1000$$

（2）组织LDH活力计算公式：

单位定义：每克组织蛋白37℃与基质作用15min，在反应体系中产生1μmol丙酮酸为1单位。

$$\text{组织中LDH活性（U/gprot）} = \frac{\text{测定OD值-对照OD值}}{\text{标准OD值-空白OD值}} \times \text{标准品浓度（2mmol/L）} \div \text{匀浆蛋白浓度（gprot/ml）}$$
