# Supplementary material for: Rat Bone Mesenchymal Stem Cell-Derived Exosomes Loaded with miR-494 Promoting Neurofilament Regeneration and Behavioral Function Recovery after Spinal Cord Injury
Source: Oxid Med Cell Longev. 2021 Oct 1;2021:1634917. doi: 10.1155/2021/1634917 (PMC8501401; doi:10.1155/2021/1634917)
Supplement: Supplementary 6 — Supplement 6: SOD test kit instructions. [file 1634917.f6.pdf]

总超氧化物歧化酶（T-SOD）测试盒（羟胺法）

50 T **WLA110a** 100T **WLA110b**

仅用于科学研究,不能用于诊断

产品信息

产品名称 总超氧化物歧化酶（T-SOD）测试盒（羟胺法）

产品概述 超氧化物歧化酶（SOD）对机体的氧化与抗氧化平衡起着至关重要的作用，此酶能清除超氧阴离子自由基（$O_2^{-}\cdot$）保护细胞免受损伤。

通过黄嘌呤及黄嘌呤氧化酶反应系统产生超氧阴离子自由基（$O_2^{-}\cdot$），后者氧化羟胺形成亚硝酸盐，在显色剂的作用下呈现紫红色，在550nm处用可见分光光度计测其吸光度。

本试剂盒可测血清（浆）、脑脊液、胸水、腹水、肾透析液、尿液、红细胞、白细胞、血小板、心肌培养细胞、肿瘤培养细胞、各种动植物组织细胞及亚细胞水平（线粒体、微粒体）中的SOD活力。

包装信息

试剂名称	WLA110a<br>（50T）	WLA110b<br>（100T）	保存条件
试剂一	20ml	40ml	4℃
试剂二	5ml	10ml	4℃
试剂三	5ml	10ml	4℃，避光
试剂四贮备液	50μlx1支	50μlx2支	4℃，避光
试剂四稀释液	5ml	10ml	4℃
试剂五	粉剂x1支	粉剂x1支	4℃，避光
试剂六	粉剂x1支	粉剂x1支	4℃，避光

保存日期 本试剂盒自订购之日起一年内有效。

注意事项

1. 每次孵育时间为40min，当室温低于20℃时孵育时间可适当延长至45min，孵育温度37℃要固定。
2. 对照管要做2支，并且放在所有测试管的中间做，取其平均值。
3. EDTA会螯合重金属酶，导致SOD活性降低，甚至测定不出，所以在使用抗凝剂收集血浆时，不能用EDTA作为抗凝剂。
4. 测试前先预试以确定最佳取样量，计算（对照管OD-测定管OD）÷对照管OD，结果应该在0.15～0.55之间，取0.45或0.48左右这一管的取样量作为最佳取样量。

试剂配制

试剂一应用液配制: 用时加入双蒸水（50T 加入40ml；100T 加入80ml），充分混匀后4℃保存一年。

试剂四应用液配制: 贮备液用双蒸水10倍稀释：再将稀释后的试剂，稀释液＝1：10比例稀释配制，现用现配，避光4℃保存。

试剂五: 加入70-80°C的热双蒸水（50T 加入37.5ml；100T 加入75ml）溶解后备用，若加热过程中水分蒸发减少，须用双蒸水补充至相应体积，避光4℃保存一年。

试剂六: 用时加入双蒸水（50T 加入37.5ml；100T 加入75ml）溶解后备用，避光4℃保存一年。

显色剂的配制：按试剂五：试剂六：冰乙酸＝3:3:2的体积比配显色剂，用多少配多少，配好的显色剂4℃避光保存三个月。

操作流程 总SOD（T-SOD）活力的测定:

试　　剂	测定管	对照管
试剂一应用液（ml）	**1.0**	**1.0**
样品（ml）	**a***	
双蒸水（ml）		**a***
试剂二（ml）	**0.1**	**0.1**

总超氧化物歧化酶（T-SOD）测试盒（羟胺法）

50 T **WLA110a** 100T **WLA110b**

仅用于科学研究,不能用于诊断

产品信息

试　　剂	测定管	对照管
试剂三（ml）	0.1	0.1
试剂四应用液（ml）	0.1	0.1
用旋涡混匀器充分混匀，置37℃恒温水浴或气浴40min。		
显色剂（ml）	2.0	2.0

混匀，室温放置10min，于波长550nm处，1cm光径比色杯，双蒸水调零，比色。

计算公式:

一、血清（浆）等液体样本中总SOD活力计算：

1、定义：每毫升反应液中SOD抑制率达50%时所对应的SOD量为一个SOD活力单位（U）。

2、血清（浆）等液体样本中总SOD活力计算公式：

$$\text{总SOD活力（U/ml）} = \frac{\text{对照OD值} - \text{测定OD值}}{\text{对照OD值}} \div 50\% \times \text{反应体系稀释倍数} \times \text{样本测试前稀释倍数}$$

二、组织匀浆中总SOD活力计算：

1、定义：每毫克组织蛋白在1ml反应液中SOD抑制率达50%时所对应的SOD量为一个SOD活力单位（U）。

2、计算公式：

$$\text{总SOD活力（U/mgprot）} = \frac{\text{对照OD值} - \text{测定OD值}}{\text{对照OD值}} \div 50\% \times \frac{\text{反应液总体积（ml）}}{\text{取样量（ml）}} \div \text{待测样本蛋白浓度（mgprot/ml）}$$
